# An experimental medicine study of the phosphodiesterase-4 inhibitor, roflumilast, on working memory-related brain activity and episodic memory in schizophrenia patients

**DOI:** 10.1007/s00213-018-5134-y

**Published:** 2018-12-08

**Authors:** James Gilleen, Yakub Farah, Cate Davison, Sarah Kerins, Lorena Valdearenas, Tolga Uz, Gez Lahu, Max Tsai, Frank Ogrinc, Avi Reichenberg, Steve C. Williams, Mitul A. Mehta, Sukhi S. Shergill

**Affiliations:** 1 Psychosis Studies, Institute of Psychiatry, Psychology and Neuroscience, Kings College London, London, SE5 8AF UK; 2 Department of Psychology, University of Roehampton, London, SW15 4JD UK; 3 Department of Forensic and Neurodevelopmental Science, IoPPN, Kings College London, London, SE5 8AF UK; 4 South London and Maudsley Hospital NHS Foundation trust, London, UK; 5 Takeda Development Center Americas, Inc., Deerfield, IL 60010 USA; 6 Eli Lilly and Company, Indianapolis, IN 46285 USA; 7 Department of Psychiatry, Icahn School of Medicine at Mount Sinai, New York, NY USA; 8 Department of Neuroimaging, IoPPN, Kings College London, London, SE5 8AF UK

**Keywords:** PDE4, Cognition, Schizophrenia, ‘Cognitive enhancement’, fMRI, Memory, ‘PDE4 inhibition’, CIAS, Roflumilast

## Abstract

**Rationale:**

Schizophrenia is associated with impairments in cognitive functioning yet there are no approved drugs to treat these deficits.

**Objectives:**

Based on animal models, we investigated the potential for roflumilast, a selective inhibitor of phosphodiesterase type 4 (PDE4), to improve cognition, which may act by increasing intracellular cyclic adenosine monophosphate in brain regions underlying cognitive deficits in schizophrenia.

**Methods:**

This study consisted of a randomised, double-blind, placebo-controlled, crossover design involving 15 schizophrenia patients. In 3 treatment periods, patients were given 8 days of placebo or one of the two doses of roflumilast (100 and 250 μg daily) with 14 days of washout between treatments. The primary endpoints were dorsolateral prefrontal cortex (DLPFC) activation during a visuospatial working memory task measured with fMRI on dosing day 8 and verbal memory and working memory performance change from baseline to day 8. Least square mean change scores were calculated for behavioural outcomes; fMRI data were analysed in SPM12 with bilateral DLPFC as regions of interest.

**Results:**

Verbal memory was significantly improved under 250 μg roflumilast (effect size (ES) = 0.77) compared to placebo. fMRI analyses revealed that increasing dose of roflumilast was associated with reduction of bilateral DLPFC activation during working memory compared to placebo, although this was not statistically significant (ES = 0.31 for the higher dose). Working memory was not improved (ES = 0.03).

**Conclusions:**

Results support the mechanistic validation of potential novel strategies for improving cognitive dysfunction in schizophrenia and suggest that PDE4 inhibition may be beneficial for cognitive dysfunction in schizophrenia.

**Trial registration:**

NCT02079844.

**Electronic supplementary material:**

The online version of this article (10.1007/s00213-018-5134-y) contains supplementary material, which is available to authorized users.

## Introduction

Patients with schizophrenia commonly experience positive symptoms such as hallucinations and delusions and negative symptoms such as lack of motivation and flattening of affect. In addition, patients also suffer from impairments in cognitive functioning, which are often profound and evident across a range of cognitive domains including learning, attention, memory and executive function (Heinrichs and Zakzanis 1998). Despite the prevalence of these cognitive deficits, there are currently no approved treatments for cognitive impairments in schizophrenia. Cognitive impairments are highly correlated with functional disability (O’Carroll 2000; Green et al. 2004) and in some domains to a greater degree than clinical symptoms (Bowie et al. 2006), so there is a significant need to develop novel therapeutic interventions to improve cognition in these patients (Gilleen et al. 2014; Gilleen 2017).

Many compounds and non-pharmacological techniques have been tested for their potential to treat cognitive deficits in schizophrenia (Wallace et al. 2011). While there have been some promising results, the dominant picture is of mixed findings and overall modest effect sizes. Pharmacological approaches include the use of agonist compounds, which may be useful in the short term, but present a theoretical limitation of adaptation of the neurotransmitter system or tolerance at the target receptor site. Compounds that indirectly alter target neurotransmitter systems may offer a more feasible approach. These include allosteric modulators, or those that inhibit enzymes, which breakdown components of neural signalling cascades.

Recently, there has also been considerable interest in the potential for inhibitors of the enzyme phosphodiesterase (PDEs) to improve cognitive functioning. PDEs are a large family of intracellular enzymes that hydrolyse the cyclic nucleotides cyclic adenosine monophosphate (cAMP) and/or cyclic guanosine monophosphate (cGMP). cAMP plays an important role as a second messenger molecule controlling multiple cellular processes (Conti and Jin 1999). Currently, 11 PDE families (PDE1–PDE11) have been identified that differ in substrate specificity, sensitivity to inhibitors and expression in different cell types. Areas of high PDE localisation are widespread, including hippocampus, prefrontal cortex, striatum and amygdala supporting a role for these enzymes in cognition (Wallace et al. 2011; van der Aart et al. 2017).

Within the PDE family, PDE4 holds specificity for cAMP and inhibition of this enzyme has demonstrated some potential for improving cognition. Rolipram, a highly selective inhibitor of PDE4 (Conti and Jin 1999), which enhances intracellular availability of cAMP (Pérez-Torres et al. 2000), has been shown to improve memory in rats (Barad et al. 1998; Hosseini-Sharifabad et al. 2012; Rutten et al. 2006; Vanmierlo et al. 2016) and reverse MK-801-induced (Zhang et al. 2000) and scopolamine-induced (Egawa et al. 1997; Zhang et al. 2000) deficits in working memory, latent inhibition (Davis and Gould 2005), as well as scopolamine-induced deficits in short-term memory (Imanishi et al. 1997; Rutten et al. 2009; Zhang et al. 2000). Rolipram attenuates PCP-induced impairments in object retrieval in monkeys (Rutten et al. 2008), and cognitive flexibility in rodents (Rodefer et al. 2012). Rolipram is a non-selective PDE4 inhibitor, and these effects may be due to the involvement of cAMP in memory as PDE4A, 4D and to a lesser extent 4C are expressed in rat hippocampal CA1 (Peng et al. 2014). Rolipram increases cAMP response in hippocampal slices and serves to facilitate persistent long-term potentiation in CA1 (Barad et al. 1998) and may do so by lowering the threshold at which LTP occurs (Bach et al. 1999).

In humans to date, there is no published assessment of the ability of rolipram to improve cognition, which may in part be due to its emetic side effects likely, in turn, due to its non-selectivity. Another PDE4 inhibitor, roflumilast, currently used to treat chronic obstructive pulmonary disease, has also shown potential to be a cognitive enhancer and has a more favourable side-effect profile perhaps attributable to its greater relative selectivity (for PDE4B/D). Although roflumilast has been used in fewer neurological studies than rolipram, the reported effects to date are favourable. Roflumilast (and rolipram) significantly reverses time-induced memory deficits in a novel object recognition task (Jabaris et al. 2015) and also enhances spatial Y-maze performance in mice (Vanmierlo et al. 2016). In healthy volunteers, Van Duinen et al. (2015) demonstrated that a single dose of 100 μg roflumilast improved verbal memory and P600 (late event related potentials) during verbal learning significantly more than placebo. Further, the same group reported that roflumilast improved verbal learning in elderly healthy volunteers (Van Duinen et al. 2017).

Cognitive deficits in schizophrenia are broad and notably include prefrontal dysfunction in addition to hippocampal-dependent memories (Heinrichs and Zakzanis 1998). Dopamine dysfunction has also been implicated in the frontally mediated deficits through the dopamine D1 receptor (Goldman-Rakic et al. 2004). D1R binding in PFC is reduced in schizophrenia, and this reduction is associated with poorer cognitive performance (Okubo et al. 1997). PDE4 can modulate dopamine signalling via its effects on cAMP, which is increased with dopamine D1 receptor agonism (Cooper 2003). Indeed, PDE4 is co-expressed with DARPP-32 in D1 receptor-positive cortical pyramidal neurons and modulates the level of D1 receptor signalling and DARPP-32 phosphorylation in the frontal cortex (Kuroiwa et al. 2012). As working memory is impaired in schizophrenia (Heinrichs and Zakzanis 1998), is functionally subserved in part by prefrontal D1 (Goldman-Rakic et al. 2004) and is disrupted by dysregulation of cAMP signalling (Taylor et al. 1999), roflumilast may be expected to improve cognition via this pathway.

In this study, we aimed to demonstrate that roflumilast, as an add-on to second-generation antipsychotics (SGAs), would improve working memory and episodic memory functioning in schizophrenia patients. We reasoned that the domains of working memory and verbal episodic memory, consistently impaired in schizophrenia, would be sensitive to modulation of PDE4 activity based on previous animal studies and the known role of cAMP in these processes. We included functional imaging of a working memory task to test the hypothesis that prefrontal cortex activity would be modulated by roflumilast, and to our knowledge, this is the first three-period crossover fMRI neuroimaging study designed to assess a cognitive enhancer in schizophrenia. We anticipated that roflumilast would enhance cognitive performance compared to placebo. Specifically, we hypothesised that there would be a significantly greater positive change in performance of verbal episodic memory (as measured by the HVLT) and working memory (as measured by the Spatial Span task), and significantly reduced dorsolateral prefrontal cortex activity during spatial working memory (as measured by the Dot Task with fMRI) following 8 days of treatment with roflumilast compared to 8 days with placebo. We also additionally explored dose-response effects of roflumilast for these cognitive and neural outcomes.

## Method

### Design

**Fig. 1:**
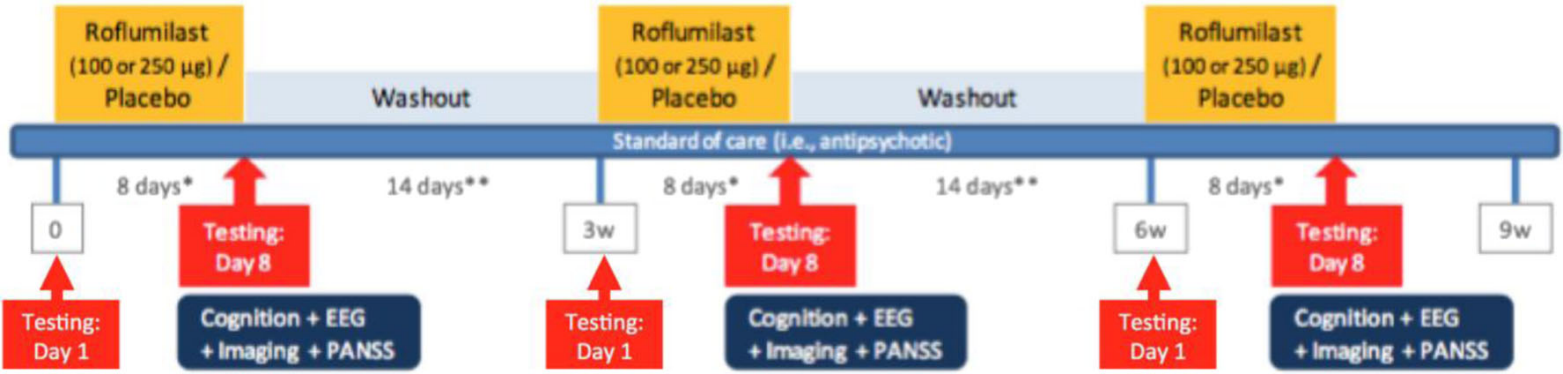
Showing study schedule

This was a phase 1, randomised, double-blind, placebo-controlled, single-site, three-period crossover study to evaluate the effect of roflumilast as an add-on to SGA in attenuating cognitive deficits associated with schizophrenia. In each of the three periods, placebo and 100 or 250 μg roflumilast were administered once daily to patients with schizophrenia for eight consecutive days to achieve steady state (Fig. 1). The time course of study participation was consistent and conformed to Fig. 1 for all participants. All patients received all doses, and the treatment order was determined by random allocation to one of three treatment sequences according to a Latin Square design (ABC, BCA or CAB), with a 2-week wash-out between phases (see Table 1).

**Table 1:** Treatment sequences

Sequence	Period 1	Period 2	Period 3
ABC (*N* = 5)	Placebo + SGA	Roflumilast 100 μg + SGA	Roflumilast 250 μg + SGA
BCA (*N* = 5)	Roflumilast 100 μg + SGA	Roflumilast 250 μg + SGA	Placebo + SGA
CAB (*N* = 5)	Roflumilast 250 μg + SGA	Placebo + SGA	Roflumilast 100 μg + SGA

Cognitive testing with the Hopkins verbal learning test-revised (HVLT-R) and spatial span (SS) from the MCCB MATRICS test battery (Nuechterlein et al. 2008) was performed on the first (pre-drug on D1) and last day (D8 post-drug) *of each phase* to assess change in cognition, and fMRI was conducted on the eighth day of each phase (see fig. 1). One week *prior* to the first drug or placebo testing phase, all participants underwent two practice training sessions for the cognitive tests and an fMRI familiarisation session. Prior task administration is a standard approach to minimise, or ‘wash-out’, practice or learning effects. These effects were further reduced by using the five available parallel versions of the HVLT-R (forms 1, 3, 4, 5 and 6), and the order of HVLT form administration was randomised and counterbalanced across participants. The DOT task trial-set is randomly generated with each test administration. For the spatial span task, no parallel versions exist however as there was at least 7 days between assessments trial-specific retention (learning effects) would not be expected to occur for such complex spatial sequences. There were three outcome measures: change in accuracy on the HVLT-R, span on the SS and neural activity change during a spatial working memory test (the Dot Task (Marquand et al. 2011)) as measured with fMRI. Performance on the Dot Task was trained to achieve a 70% correct threshold, and only correct trials (hence *successful* working memory) were included in the analysis. FMRI was carried out while subjects performed the Dot Task in the MR scanner while the other cognitive tasks, the HVLT and spatial span task, were performed just prior and outside the scanner.

This study took place at the Institute of Psychiatry, Psychology and Neuroscience, Kings College London. This research was conducted in accordance with the protocol procedures complied with the Declaration of Helsinki, the International Conference on Harmonisation (ICH) E6 GCP guidance, and all applicable local or regional regulatory requirements. The study was pre-registered with clinicaltrials.gov (identifier: NCT02079844), and hospital ethics approvals were given by NRES Committee South Central Berkshire (reference 12/SC/0443).

### Participants

Patients who had consented to be contacted about participating in research were recruited from local outpatient services. Following the study inclusion/exclusion criteria, men or women, aged 18 to 60 years, who met Diagnostic and Statistical Manual of Mental Disorders, 5th Edition (DSM-5), criteria for schizophrenia, and who were receiving stable doses of second-generation antipsychotics for at least 2 months prior to screening (see supplementary materials for inclusion/exclusion criteria) were eligible to take part. Twenty-one patients were consented and randomised to one of the treatment groups. Participants were randomised to one of three treatment sequences according to a blinded computer-generated allocation schedule. Six did not complete the study: five participants withdrew without reason, and one was unable to attend dosing and so was withdrawn. Demographics and clinical information for the 15 participants who completed are shown in Table 2, and additional information on participants is shown in supplementary materials. Only data from completers is reported. Seven participants were receiving olanzapine (2 at 20 mg, 2 at 10 mg, 1 at 15 mg, 1 at 10 mg, 1 at 2.5 mg), four were receiving aripiprazole (2 at 20 mg, 1 at 15 mg, 1 at 10 mg), one quetiapine 300 mg, one paliperidone 50 mg and two risperidone depot 25 mg.

**Table 2:** Participant demographic and clinical information

	Mean (*SD*)
Age (years)	40.5 (*10.5*)
Gender (male/female)	10:5
Ethnicity	9 Black British, 4 White British, 2 British Asian
Age of illness onset (years)	32.7 (*6.2*)
Age of illness duration (months)	170.9 (*116.3*)
Time on current antipsychotic treatment (months)	74.1 (*56.6*)
Baseline PANSS Total	59.9 (*16.1*)
Baseline PANSS Positive	14.3 (*4.3*)
Baseline PANSS Negative	15.1 (*5.2*)
Baseline PANSS General	30.5 (*8.1*)

### Dose

It has been demonstrated that single doses of roflumilast 100 μg (but not 300 or 1000 μg) administration improves episodic memory in both healthy volunteers; with the same pattern—single dose 100 μg (but not 300 or 1000 μg)—showing significant effects on P600 values with EEG (24,25, 40). In following, 100 and 250 μg in multiple dosing to achieve steady-state condition were chosen for this study to explore the effective dose window in patients with schizophrenia. Dosing on all days took place at site in front of a research team member to ensure compliance—confirmed post hoc via pharmacokinetic/metabolite analysis of blood samples. Plasma concentrations, roflumilast exposure and safety data are reported in supplementary materials. Both doses were compared to placebo. Blinding and randomisation procedures were conducted by Quintiles CRO Ltd. Blindings for this study were not broken for any participant.

### Measures

Two memory subtests from the MATRICS Consensus Cognitive Battery (MCCB (Nuechterlein et al. 2008)) were administered:

#### The Hopkins verbal learning test-revised (Brandt and Benedict 2001)

The Hopkins verbal learning test-revised (HVLT-R) is a subtest of the MATRICS Consensus Cognitive Battery (MCCB (Nuechterlein et al. 2008)). It assesses verbal memory and consists of a list of 12 words from 3 taxonomic categories. The words were presented orally, and the participants are asked to recall as many words as possible after each of three trials. The key outcome variable for the HVLT-R was the total number of correct responses over three trials. The test was administered on day 1 (pre-drug) and day 8 (post-drug), and each drug period made use of the alternate HVLT-R forms from the MATRICS to avoid practice effects.

#### The spatial span test (Wechsler memory scale 3rd edition; Wechsler 1997)

The spatial span test assesses working memory. Participants are presented with a board containing blue blocks arranged in an irregular pattern. The rater taps out a pattern on the blocks and participants are tasked with tapping the same pattern in response. Trial spans become longer in length and also consist of both forward and reverse patterns, the latter requiring patients to tap in the reverse order to the administrator. Scores are the sum of the correct responses in both the forward and backward trials.

### fMRI

#### The DOT task

**Fig. 2:**
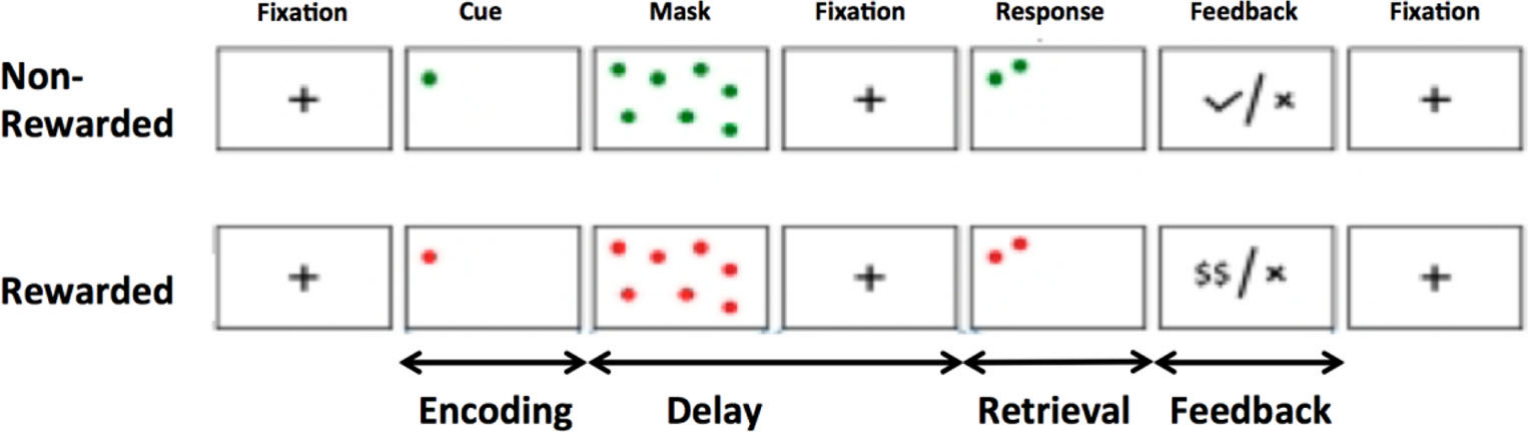
Showing task schematic for the Dot Task

In the DOT task (see Fig. 2), a rewarded delayed-response working memory task (Marquand et al. 2011), participants are instructed to remember the spatial location of a target stimulus (a dot) relative to a fixation cross after a delay period, which includes a noise mask (numerous moving dots). Half of the 40 trials carried a monetary reward, indicated by the colour of the stimulus, and the order of the trials was randomised and counterbalanced across subjects. In each trial, the target stimulus was presented for 750 ms (encoding), followed immediately by a mask for a further 750 ms to disrupt visual iconic memory; after a 7- or 9-s interval (delay), the target stimulus and a distracter stimulus are presented, and participants indicated which of the stimuli matches the target location by a left- or right-button press on a two-button response box (retrieval; 2 s). Feedback indicating success or failure is provided at the end of each trial. Patients received training on the task to ensure they understood the instructions, were able to respond appropriately and could achieve a threshold performance of 70% correct trials—well above chance. Only correct trials were used in the analysis as we were interested in successful spatial working memory (evident from correct trials only)—hence performance changes per se were not examined, only BOLD response changes during spatial working memory. FMRI was only conducted on D8 (post-drug), and these sessions were compared between drug arms. Strict criteria were used for acceptance of valid data available in all three sessions.

#### Symptom measures

Symptoms were assessed at days 1 and 8 using the PANSS (Kay et al. 1987), although the study design was focussed on, and powered for, the cognitive outcomes.

### Statistical analysis

Statistical analyses were performed to examine the effect of each dose of drug and placebo on change in cognitive test scores from day 1 (baseline) to day 8 (post-drug assessment) for the endpoints involving cognitive test results relative to change in the placebo phase from day 1 (baseline) to day 8 (post-drug assessment), and by day 8 comparison for the fMRI endpoints. Data processing and voxelwise brain image analysis was performed by the investigators, with the analysis of the primary endpoints performed by an independent statistician.

#### Cognition

Pairwise comparisons were made between each roflumilast dose level and placebo using LS means estimated via analysis of variance (ANOVA) with sequence, period and treatment as fixed effects and subject nested within sequence as a random effect. The effect size for each roflumilast dose level was calculated using Cohen’s d from which an effect size of 0 to 0.2 is considered small, 0.3 to 0.7 is considered moderate, and ≥ 0.8 is considered large.

#### fMRI

##### MRI data acquisition

We collected a high-resolution T1-weighted image on one session for each participant, for normalisation to a standard space (TR = 7312 ms, TE = 3.016 ms, FoV 270 mm, voxel size 1.05 × 1.05 × 1.2 mm, 196 slices, TI = 400 ms). The functional time series used the following parameters (TR = 2000 ms, TE = 30 ms, FoV 211 mm, voxel size 3.75 × 3.75 × 3.3 mm, 39 slices).

##### fMRI and roflumilast

The fMRI task data acquisition started 5 h after day 8 dosing. The Tmax is up to 3 h after ingestion of a single dose and its *N*-oxide metabolite demonstrates a plateau-like Tmax after about 8 h. The median effective half-life of roflumilast is 17 h. Steady-state plasma concentrations following repeated dosing with 500 μg Roflumilast is estimated to be reached after approximately 4 days (83 h) for roflumilast and after 6 days (148 h) for the *N*-oxide metabolite. Roflumilast and its *N*-oxide show linear repeated-dose pharmacokinetics over the dose range of 250 to 1000 μg. Thus, assessment after 8 days of treatment ensured steady-state plasma concentrations required for cognitive testing.

##### fMRI modelling

Preprocesing steps are reported in the Supplementary materials. Single-subject, single-session fixed-effects GLM analysis was conducted to model responses during the DOT task. The DOT task consists of three parts: encoding, delay and retrieval, and additionally, trials were either rewarded or unrewarded. Thus, the following regressors were modelled: non-rewarded encoding and delay, non-rewarded retrieval, non-rewarded feedback, rewarded encoding and delay, rewarded retrieval and rewarded feedback. Because of the proximity in time and the brief, fixed presentation time of the cues, the encoding and delay periods were modelled together. The six movement regressors from the motion correction were also included in the model. A high pass filter of a period of 128 s was applied to the model to eliminate low-frequency signals such as scanner drift. For the purposes of reporting the data here, contrasts for the encoding and delay period were combined across the rewarded and non-rewarded trials as the primary outcomes were based on working memory-related activity.

The individual contrast maps from the first level were entered into a second level, random-effects analysis with subject as the random effect and session (drug/placebo) as a within-subject effect. Linear contrasts were used to describe the main effect of task across all drug conditions and the effect of roflumilast on the a priori defined region of interest.

fMRI performed well across participants in relation to the cognitive task and data collected, although data from some were removed due to poor quality. Quality was assessed for the fMRI data by visually inspecting coverage within the field of view and calculating movement. Excessive movement (> 1 voxel > 20% volumes) in any one session resulted in removal of the participant from the fMRI analysis. A high response rate was required for the task (> 60%), and no participant was removed due to low response rate.

#### Regions of interest (ROI)

A dorsolateral prefrontal cortex (DLPFC) ROI was chosen due to its known involvement in working memory (Curtis and D’Esposito 2003) defined as 6-mm spheres centred on the peak co-ordinates from Marquand et al. (2011) (Left − 43, 33, 25, Right 38, 44, 32), which utilised the same task.

## Results

### HVLT-r

LS mean HVLT total scores on day 8 (compared to Day 1) were increased with roflumilast 100 and 250 μg (0.27 (SE = 1.06) and 1.07 (SE = 1.08), respectively) and decreased with placebo (− 1.67, SE = 1.08) (see Table 3). LS mean differences in change from baseline between high-dose roflumilast and placebo were statistically significant (*p* = .044, ES = 0.77 (large effect size)), but not between low-dose roflumilast and placebo (*p* = .14, ES = 0.55 (medium effect size)). We also conducted an ITT (Intention To Treat) analysis which revealed the following effects: 100 μg (*N* = 17) vs placebo (*N* = 16): ES = .45 (95% CI interval = − 0.25 to 1.14); and 250 μg (*N* = 16) vs placebo: ES = 0.55 (95% CI interval = − 0.16 to 1.26). These differences were not significantly different.

**Table 3:** Mean behavioural performance scores (sds) for the cognitive tasks at each drug level

Dose	Placebo		Low dose		High dose	
Day	D1	D8	D1	D8	D1	D8
HVLT-R	21.93 (*4.77*)	20.20 (*4.25*)	20.33 (*5.41*)	20.60 (*5.53*)	19.53 (*6.55*)	20.60 (*6.06*)
Spatial Span	14.67 (*3.96*)	14.47 (*4.14*)	14.47 (*3.83*)	14.40 (*3.72*)	14.73 (*3.90*)	14.60 (*3.78*)
Dot Task percent accuracy^a^		69.41 (*16.39*)		69.01 (*19.15*)		70.71 (*15.14*)

*D1* day 1, *D8* day 8, *HVLT* Hopkins verbal memory test

^a^Dot Task was only done at D8

Post hoc examination of the three HVLT trials revealed that performance on the high-dose roflumilast and placebo was maximally different on the third and last recall test of the three HVLT-R word list repetitions (effect size for trial 1 = 0.32, trial 2 = 0.47, trial 3 = 0.73).

### Spatial span

LS mean total score change from D1 to day 8 with roflumilast 100 and 250 μg were 0.07 (SE = 0.51) and − 0.13 (SE = .52), respectively, while scores decreased with placebo (− 0.20, SE = 0.52). Change under both roflumilast doses were not significantly different to placebo (low dose *p* = .74; high dose *p* = .93). An ITT analysis (placebo: *N* = 16, 100 μg *N* = 17, and 250 μg *N* = 16) also revealed no significant drug effects vs placebo (100 μg (*N* = 17) vs placebo (*N* = 16): ES = 0.11 (95% CI interval = − 0.57 to 0.79), and 250 μg (*N* = 16) vs placebo: ES = 0.06 (95% CI interval = − 0.63 to 0.76).

### fMRI

#### The DOT task

**Fig. 3:**
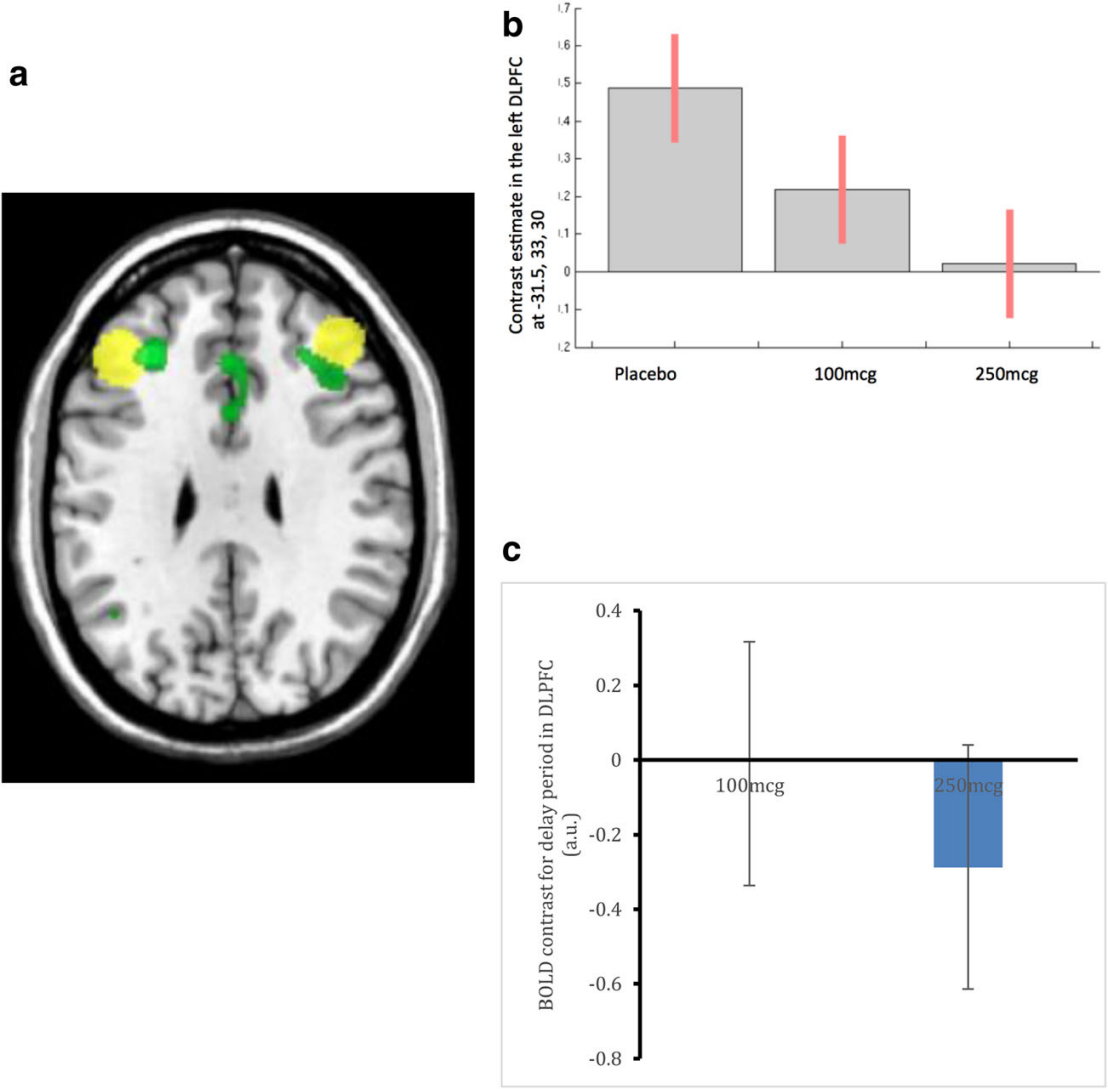
Showing DOT task fMRI results. **a** showing brain regions where activity was lower with 250 μg (green; a priori ROIs in yellow). **b** showing extracted beta coefficients of activity for the peak voxel within the a priori ROI at each drug dose (*n* = 10). **c** is a histogram showing the LS mean difference from placebo and 95% confidence intervals for all 13 participants who completed the DOT task

Complete data were acquired for ten participants. Participants were as evenly distributed across the treatment orders as possible. For the comparison of placebo with 100 and 250 μg, there was change of activity with effect sizes of − 0.03 and − 0.671, with *p* values of 0.948 and 0.154 respectively. A voxelwise analysis was additionally conducted, and this again showed a reduction in activity from placebo to 250 μg roflumilast (shown in Fig. 3a, b) although this did not survive FWE-correction for multiple comparisons in the DLPFC. An additional exploratory whole-brain analysis was performed, and this revealed that no other brain regions were significantly affected by roflumilast; thus, no changes outside the DLPFC were observed. A separate analysis of the same data but including all 13 patients who completed the task (at any time point) showed effect sizes of − 0.006 for 100 μg (*p* = .98) and 0.500 for 250 μg (*p* = 0.082). The mean difference in BOLD response for the low and high doses is illustrated in Fig. 3c. While these analyses are in line with our prediction of reduced activity in the DLFPC with roflumilast, the differences were not significant. Post hoc analyses of the rewarded and unrewarded trials separately showed the same pattern of results: less activity associated with the high dose, most with placebo and the low dose producing intermediate activity. Behaviourally, as participants were trained to perform well on the task (to a threshold of ~ 70%) and only correct trials were used in the analysis performance was intended to be matched across treatment arms. As shown in Table 3, this was the case, and differences were not statistically different.

#### Symptom ratings

PANSS ratings did not change from **d**ay 1 to 8 for either dose of drug on either the positive or negative scale (*p*s > 0.434).

## Discussion

This study tested the hypothesis that the addition of roflumilast, a PDE4 inhibitor, would improve cognitive function and modulate frontal brain activity in patients with schizophrenia treated with atypical antipsychotics. The performance on verbal learning from the HVLT was significantly improved with the higher dose of roflumilast compared to placebo; however, working memory measured with the spatial span test did not show improvement. Reductions in PFC activity during working memory were evident with increasing doses of roflumilast in bilateral DLPFC, and the effect size for the higher dose was 0.5.

### Verbal learning

There is considerable preclinical evidence suggesting that inhibition of the PDE4 enzyme improves cognition, particularly memory processes (Bach et al. 1999; Barad et al. 1998; Hosseini-Sharifabad et al. 2012; Rodefer et al. 2012; Rutten et al. 2006; Rutten et al. 2008; Rutten et al. 2009; Vanmierlo et al. 2016; Zhang et al. 2000). Performance on verbal list learning was significantly enhanced by roflumilast in patients with schizophrenia—and these effects were greatest on the third and final learning presentation. Strikingly, Van Duinen et al. (2018) have also recently reported improvement in verbal learning performance following a single-dose 100 μg of roflumilast in healthy adults—and also showing greatest effects on the third trial in healthy adults as demonstrated here. The P600 peak as measured by EEG during verbal learning was also significantly increased, again only on the third trial at 100 μg, not at 300 or 1000 μg, of roflumilast. The authors suggest these findings may indicate that roflumilast affects information processing during memory (word) encoding.

There is now emerging and converging evidence that PDE4 inhibition can enhance memory via its action on long-term potentiation (LTP). Rolipram specifically induces late-phase LTP (L-LTP) not early-phase LTP (E-LTP) in rat hippocampus (Barad et al. 1998), and similarly specifically modulates late-long-term memory (LTM) not early-LTM (Rutten et al. 2009). It has been proposed that increasing cAMP signalling may improve memory by raising the probability that long-lasting synaptic plasticity occurs (Barad et al. 1998; Blokland et al. 2012). In line with this, mice with impaired long-term object recognition, due to being carriers of truncated CREB-binding protein, show reversal of deficits with rolipram (Bourtchouladze et al. 2003). In humans, the hippocampus is active during memory, which requires longer-term storage, including verbal learning (Johnson et al. 2001). Thus, as potential for LTP will be maximal with an increased number of stimuli presented over a time period, elevation of L-LTP/L-LTM by roflumilast is a potential neurobiological mechanism by which verbal learning performance improvements may occur.

### Working memory

Roflumilast was associated with reduced cortical demands during spatial working memory assessed using fMRI. This modulation was dose-dependent with a medium effect size for the higher dose, suggesting improved neural efficiency. We proposed that roflumilast would improve working memory as PDE4 inhibition increases intracellular cAMP levels in striatum and frontal cortex. These alterations increase dopamine synthesis and turnover in striatum and enhance the dopamine D1 receptor/PKA/DARP-32 signalling cascade in frontal cortex. Inhibition of PDE4 by rolipram increases dopamine D1 receptor signalling in cortical pyramidal neurons (in mouse) in frontal cortex (Kuroiwa et al. 2012), and dopamine D1 receptor signalling is known to improve working memory performance in rodents and non-human primates (Goldman-Rakic et al. 2004). The preclinical evidence also indicated that PDE4 inhibition reverses MK-801-induced (Vanmierlo et al. 2006) and scopolamine-induced (Egawa et al. 1997) deficits in working memory. As an example, activation of NMDA receptors produces a CA2-dependent increase in cAMP that may play a role in the induction of LTP in CA1 of the hippocampus (Zhang et al. 2000).

Roflumilast did not, however, lead to significant change in cognitive performance on spatial span compared to placebo at either dose. The pattern of data in the prefrontal cortex fits with previous neuroimaging studies of catecholaminergic stimulant medication (e.g., Mehta et al. 2000) with reductions in task-related activity accompanied by improvements, or even lack of significant change in performance. Future studies could investigate the specific PDE4 neurotransmitter interactions, which may account for pro-cognitive effects of PDE4 inhibition in working memory, changes in neural efficiency, and other cognitive processes.

### Limitations

The main limitation of this mechanistic study was the relatively low subject numbers, partly due to attrition because the patients found the frequency and complexity of assessments within the study somewhat demanding. As this study is exploratory in nature, the sample size was not based on statistical considerations. The sample size is based on the desire to gain adequate efficacy data on the primary and secondary outcomes as well as on published literature reporting pro-cognitive effects of drug candidates using the same pharmacological model and similar study. However, using a within-subject, crossover design rescues some statistical power by reducing degrees of freedom.

A primary goal of the study was to provide evidence of improved cognitive function following PDE4 inhibition in patients for the first time, and to assess the underlying impact on frontal brain activation, In both respects, it was successful and demonstrates the utility of combining cognitive and imaging markers in studies of novel mechanisms in patient populations. Since the outcomes were only measured at one time point, we are unable to determine if the drug effects are in fact greater earlier after an acute medication administration, or alternatively, if longer-term treatment leads to more robust effects in patients.

The delayed-response working memory fMRI task was based on one of the most widely used working memory paradigms, is sensitive to pharmacological manipulation and incorporated reward and no-reward conditions in order to parallel both the human and animal research into working memory processes. This also allows for secondary analyses to examine the role of reward; however, there was no differential effect of roflumilast on reward. This could reflect either a real lack of effect, or poor sensitivity due to the low sample size.

Additionally, for the purposes of this initial trial, we minimised the number of endpoints and chose to examine only the DLPFC during administration of the DOT task as this aligned with the predicted effects in schizophrenia and the predicted effects of PDE4 inhibition on the prefrontal activation seen during the encoding and delay periods on the task; however, subsequent work could investigate additional brain regions.

The age at illness onset ranged from 17 to 44 years, and the mean age of participants was 32 years old which is relatively later than in other patient studies. An older average age of patients is, however, more likely to be expected in drug trials as clinical and medication stability are inclusion criteria, and this is somewhat more likely in older patients. Despite this, the higher mean age may also be due to the relatively small sample size and could potentially limit the generalizability of the findings to younger onset samples.

The significant mean score differences between high-dose roflumilast and placebo on the HVLT-R were contributed to in part by a decline over time in the placebo arm. Despite this, the trend from placebo to high dose was significantly greater improvement with the high dose than placebo. Decline in the placebo group may additionally be attributable to study fatigue, which may be modified in the treatment periods.

## Conclusions

Individuals with schizophrenia have significant cognitive deficits across many domains of functioning including memory, learning and working memory. Improvements in cognitive functioning are associated with better overall outcomes in individuals with schizophrenia (Bowie et al. 2006), yet there are no currently approved drugs for the improvement of cognition in schizophrenia. This study investigated the potential for roflumilast, a PDE4 inhibitor, to provide pro-cognitive effects in patients with schizophrenia concurrently treated with second-generation antipsychotics. The results showed that high-dose roflumilast significantly improved verbal learning relative to placebo. There was also a trend for roflumilast decreasing activation in the DLPFC during working memory suggestive of an impact via improving neural efficiency. These results are in line with previous studies with experimental animals indicating that PDE4 inhibition may improve memory function through its impact on intracellular mechanisms. Memory may be enhanced via the effects of cAMP on two systems—one, fast-acting on prefrontal neurotransmission to improve online memory processes, and a second, enhancing the capacity of L-LTP to encode learning targets (Kuroiwa et al. 2012). In summary, this clinical study provides support for increasing the use of combined neuroimaging and cognitive testing to validate models developed in experimental animals. We suggest that this research also supports further evaluation and clinical trials of this well-tolerated, off-patent and widely prescribed treatment as a potentially repurposed medication from respiratory medicine to neuropsychiatry. It should also be noted for subsequent studies that cAMP gradients and responses are highly varied among PDE isoforms (Fertig and Baillie 2018); thus, future work may better couple specific targeting of PDE4 subtypes with cognitive effects, potentially avoiding the need for multiple days of dosing to achieve steady state, as well as potential optimisation of side-effect profiles.

## Electronic supplementary material

ESM 1 (DOCX 82 kb)
