# Supplementary material for: An experimental medicine study of the phosphodiesterase-4 inhibitor, roflumilast, on working memory-related brain activity and episodic memory in schizophrenia patients
Source: Psychopharmacology (Berl). 2018 Dec 8;238(5):1279–89. doi: 10.1007/s00213-018-5134-y (PMC8062361; doi:10.1007/s00213-018-5134-y)
Supplement: Supplementary file 1 — (DOCX 82 kb) [file 213_2018_5134_MOESM1_ESM.docx]

**Supplementary Materials**

**Summary**

The supplementary materials consist of this single word document, the contents of which are:

- 1. Patient Inclusion / Exclusion Criteria
  2. Participant drop-out
  3. Plasma concentrations of Roflumilast
  4. Roflumilast exposure
  5. Safety
  6. fMRI preprocessing

**1.1 Patient Inclusion / Exclusion Criteria**

Patients had to (a) be on stable doses of second generation antipsychotics for at least 2 months prior to screening (b) score ≤4 on the Conceptual Disorganization, Hallucinatory Behaviour and Unusual Thought Content items and all negative subscale items from the Positive and Negative Symptom Scale (PANSS (33)); and (c) have cognitive impairment as judged by the investigator. Patients were excluded if they had uncontrolled, clinically significant neurological abnormalities; a positive urine drug result for drugs of abuse at screening or day 1 of each treatment period; a history of drug abuse (defined as any illicit drug use) or alcohol abuse within 6 months prior to the screening; treatment with clozapine in the last year; and if they were unwilling to agree to abstain from alcohol and illicit drug use throughout the study.

**1.2 Participant drop-out**

Thirty-one people were consented in total, and of those, twenty-one were randomised to the study. Thus ten participants were ineligible to take part. The reasons were:

- BMI too high (N = 4)
- Positive urine drug screen (N = 2)
- Ineligible due to unstable dosing on SGA criteria (N = 3)
- Withdrew consent (N = 1)

Six participants did not complete the study having started. The reasons were as below. Side-effect monitoring and final visit assessments did not support adverse effects being responsible for these drop-outs.

- Unable to attend dosing (N = 1)
- Withdrawal without reason (N = 5)

**1.3 Plasma concentrations of Roflumilast**

Blood samples were collected from all subjects prior to dosing on Day 1 and at specific time points on Day 8 postdose. Plasma concentrations of roflumilast and its metabolite N-oxide roflumilast were determined using a validated liquid chromatography-tandem mass spectrometry assay with a validated range of 0.5 to 1000 ng/mL for both analytes.

The pharmacokinetics (PK) of roflumilast and N-oxide roflumilast were characterized using previously described population PK models (ie, a 2- compartment model with linear absorption and elimination and a 1-compartment model with zero-order absorption and linear elimination, respectively) (Lahu *et al*., 2010). Model-based simulations were subsequently performed to project the individual time-course of roflumilast and N-oxide roflumilast over the treatment period using the empirical Bayesian estimates derived from the model. The population PK analysis and simulations were conducted using the first-order conditional estimation method with η-ε interaction in NONMEM Version 7.1.2.

**1.4 Roflumilast exposure**

PK models for roflumilast and N-oxide roflumilast were developed using PK data in subjects with schizophrenia, starting with previously published models. Slight modifications to the published models include estimation of certain key parameters (absorption rate constant for roflumilast and apparent clearance and central volume of distribution for N-oxide roflumilast), improved the fit of the models to the study data. Otherwise, the pharmacokinetic data from this analysis were generally consistent with the historical data in healthy subjects and subjects with COPD published in the literature (35). For roflumilast, the mean Cmax (ng/mL) values at 100 and 250 µg were 1.32 (sd = 0.33) and 3.24 (sd = 0.81), respectively, and mean AUC (ng hr/mL) values at 100 and 250 µg were 9.11 (sd = 4.27) and 22.52 (sd = 10.98) respectively. For roflumilast N-oxide, the mean Cmax (ng/mL) values at 100 and 250 µg were 4.81 (sd = 2.01) and 11.81 (sd = 5.11), respectively, and mean AUC (ng hr/mL) values at 100 and 250 µg were 102.54 (sd = 45.38) and 252.12 (sd = 115.81) respectively, close to the values reported in a recent publication by Van Duinen *et al.* (2018).

***1.5 Safety***

Collection of AEs commenced from the time the participant was first administered study medication (Day 1 of Period 1). Routine collection of AEs continued throughout the trial up to and including the follow-up visit after all three phases of the study were complete and these recorded on study CRFs. AE assessment was part of the study’s routine scheduled study activities, but patients could also report AEs themselves to the study team at any point. Multiple doses of roflumilast 100μg and 250μg (QD for 8 days) were well tolerated in this study. The percentage of subjects who had any TEAE was similar after administration of roflumilast 100μg and roflumilast 250μg (41.2% and 47.4%, respectively) and higher after administration of placebo (56.3%). Only headache and nasopharyngitis occurred in 2 or more subjects after administration of roflumilast; headache occurred in 2 subjects after administration of roflumilast 250μg; and nasopharyngitis occurred in 2 subjects after administration of roflumilast 100μg. All TEAEs were mild or moderate in intensity. No deaths occurred. One subject had an SAE (suicide attempt) leading to discontinuation of study drug - however inspection of treatment order after unblinding at the end of the study indicated this occurred during the placebo phase; and, additionally, the patient’s significant negative life experiences that the research team were aware of at the time that, in our judgment, most likely explained the suicidality. No other significant adverse events occurred. No pregnancies or overdoses were reported.

**1.6 fMRI Preprocessing**

Data were pre-processed using Statistical Parametric Mapping (SPM 12; Wellcome Trust Centre for Neuroimaging, London, UK; <http://www.fil.ion.ucl.ac.uk/spm>) according to the following analysis pipeline. The T1-weighted images were first segmented, and then DARTEL was utilised to create a group template. Slice-timing correction was applied to the functional time-series, followed by within-subject rigid-body realignment. This process involves realignment of all of the image volumes to the first imaging volume and then to the mean image. Within-subject, between-modality coregistation, utilising affine transformation of the mean time series images to the same subject’s T1-weighted image in native space. This co-registration was then applied to all of the image volumes in the time-series. The data were then spatially normalised using the DARTEL flow fields from each subject to warp the coregistered functional images to the MNI template space. Data were smoothed using an 8mm FWHM kernel to improve signal to noise ratio. Realignment parameters and regressors for CSF and white matter were used as nuisance regressors to account for head motion and shared variance in the analysis.
